# Challenges for drug checking services in Scotland: a qualitative exploration of police perceptions

**DOI:** 10.1186/s12954-022-00686-6

**Published:** 2022-09-23

**Authors:** Danilo Falzon, Elizabeth V. Aston, Hannah Carver, Wendy Masterton, Bruce Wallace, Harry Sumnall, Fiona Measham, Emma Fletcher, Rosalind Gittins, Saket Priyadarshi, Tessa Parkes

**Affiliations:** 1 Salvation Army Centre for Addiction Services and Research, Faculty of Social Sciences, University of Stirling, Stirling, UK; 2 School of Applied Sciences, Edinburgh Napier University, Edinburgh, UK; 3 School of Social Work, University of Victoria, STN CSC, PO Box 1700, Victoria, BC Canada; 4 Public Health Institute, Liverpool John Moores University, Liverpool, UK; 5 Department of Sociology, Social Policy and Criminology, University of Liverpool, Liverpool, UK; 6 NHS Tayside, Dundee, UK; 7 Clinical Department, Humankind Charity, Durham, UK; 8 NHS Greater Glasgow and Clyde, Glasgow, UK

**Keywords:** Drug checking, Policing, Criminalisation, Community based, Harm reduction, Stop and search, Public health, Scotland, Qualitative, Drug use

## Abstract

**Background:**

The impact of policing practices on the engagement of people who use drugs (PWUD) with harm reduction services is well evidenced. Although the police have traditionally taken an enforcement role in responding to drug use, it is increasingly clear that they can play an important part in multiagency delivery of harm reduction interventions. Despite this, there have been no studies exploring police officer perceptions of drug checking services (DCS), which provide analytical testing of client drug samples alongside harm reduction support and advice.

**Methods:**

Semi-structured interviews were conducted with 10 police officers to explore the policing and legal challenges which could be encountered in the delivery of DCS in Scotland.

**Results:**

Participants expressed general support for DCS and described this support as part of a wider organisational shift towards public health-oriented policing. Participants also discussed different potential approaches to the policing of areas surrounding DCS including: formal limits on police presence around the service and/or stop and search powers in relation to personal possession; the effective decriminalisation of personal possession within a specified boundary around the service; and informal agreements between local divisions and DCS outlining expected policing practices. Any formal limitation on the capacity of police officers to respond to community concerns was viewed as problematic and as having the potential to erode public confidence in policing. Participants also highlighted the potential for frontline officers to utilise discretion in ways which could undermine public health goals. Legislative change, or national strategic guidance from relevant stakeholders, was seen as a means of providing ‘cover’, enabling local divisions to support the operation of drug checking.

**Conclusions:**

Despite a small sample of participants, this study summarises key challenges to be addressed in the implementation and operation of DCS in Scotland, and more widely. The paper concludes with suggested opportunities to develop approaches to policing that can facilitate rather than impede implementation and operation of these services.

## Background

Drug checking services (DCS) can be defined as an intervention ‘whereby service users receive test results for a substance of concern submitted for forensic analysis as part of a harm reduction consultation’ [1]. The number of DCS globally is growing rapidly, as drug checking is increasingly recognised as a valuable harm reduction and market monitoring intervention [2–4]. Community-based DCS operate in a range of legal contexts, with varying levels of protection and support from legislation, and national and local police forces [5–7]. There are two primary, inter-related, issues in relation to the legal challenges facing DCS. The first relates to the legality of the service itself, and the legal protection afforded to staff. Services operate with different levels of protection in this regard, ranging from explicit legislation or ‘legal exemptions’ protecting their operation, to varying degrees of informal, tacit acceptance by local police forces [5–11]. The second relates to the protection of clients from being charged for drug offences by police when entering, leaving, and travelling to services [12]. Whilst DCS have received legal exemptions protecting their operation in some countries, such as Canada and the UK [10, 13], such exemptions relate to the operation of the service and its staff, providing no explicit protection to clients attempting to access such services. Indeed, few DCS operate in contexts providing explicit legal protection for clients [1, 5, 9, 13, 14]. One clear exception is the Netherlands, where there is an agreement with the public prosecutor that people will not be prosecuted for possession when trying to access DCS [15]. However, generally, DCS rely on police discretion and informal agreements, leaving prospective clients potentially vulnerable to criminalisation [5, 6, 8]. Despite the lack of explicit legal protection for those accessing the service, there is little in the literature exploring policing practices in the vicinity of DCS and limited available information on how such practices vary across jurisdictions.

There is strong evidence that enforcement-based policing practices in relation to drug possession for personal use can negatively impact upon health outcomes for people who use drugs (PWUD), and can reduce engagement with harm reduction supports and services due to fear of criminalisation [16–23]. ‘Enforcement-based’ policing practices in relation to drug possession can be defined as those which aim to deter and punish drug possession through the strict enforcement of drug laws [24]. Such an approach is characterised by high levels of stop and search and patrols and a range of further tactics in spaces where there are concerns over public order [16, 18, 25]. Such practices have been shown to disproportionately impact marginalised groups such as those experiencing homelessness and those engaged in open drug markets [18, 26], intersecting with factors such as race and socio-economic position [26].

Whilst there is substantial evidence on the negative impacts of enforcement-based practices on the health outcomes of PWUD, there is also increasing exploration of the ways in which policing practices may be altered to be more in line with public health goals [16, 24, 25, 27, 28]. Such an approach to policing has been termed in the literature ‘public health’ or ‘harm reduction’ policing. Although there is debate regarding the scope and characteristics of public health policing, it can be broadly defined as an approach ‘that aim[s] to reduce the adverse health, social and economic consequences of drug use, drug markets and the efforts to control them through the criminal justice system’ [24]. Such a definition encompasses a broad range of potential policing practices—from reductions in stop and search on grounds of suspected possession [15], to signposting individuals to harm reduction services [29]. However, the general thrust of such an approach is decreasing use of enforcement and criminalisation in relation to personal possession offences [16]. There is evidence of increasingly varied approaches across countries to dealing with drug use and possession [30]. Relatedly, policing, in a number of jurisdictions, is shifting from enforcement-led approaches, to an approach more aligned with public health outcomes [15, 24]. Such shifts in practice create important questions about the role and function of policing in relation to drug use, as well as about potential tensions between practices of enforcement and practices of care [24].

Debates around policing practices and the criminalisation of personal possession, and the relationship of these factors to the implementation and operation of harm reduction services such as DCS, are highly relevant to the Scottish context [31]. Scotland has the highest rate of drug related deaths in Europe [32]. In 2021 there were 1330 drug related deaths, an age standardised rate of 23 per 100,000 of the population, and an increase of approximately 250% since 2013 [33, 34]. There are growing calls from public health, political actors, researchers and activists to implement harm reduction interventions such as DCS and drug consumption rooms to address the ongoing public health crisis [31, 35–37]. Conversations concerning the implementation of both services are ongoing amongst a range of stakeholders [36–38], with the implementation of DCS currently being proposed and worked towards in three Scottish cities [39]. Policing practices in relation to DCS in Scotland are a key factor which could either facilitate engagement, through public-health aligned practice, or act as a barrier to engagement, through enactment of the enforcement-based practices outlined above. Given strong evidence that criminalisation of personal possession is contrary to harm reduction and public health goals [27, 30, 40–43], shifting practice towards a more public health-based approach holds promise for addressing the current high levels of drug related harms in Scotland, through enabling access to harm reduction services such as DCS.

Drug possession is a criminal offence in Scotland subject to the Misuse of Drugs Act 1971 and, as drug law is not a devolved power, levers of legislative change lie with the UK Government. The UK Government, despite expansion of diversionary schemes throughout England in recent years [24, 44], has stated that it does not currently support decriminalisation of personal possession—its most explicit statement to this effect being in response to the Scottish Affairs Committee’s recommendation to decriminalise possession for personal use in 2019 [45, 46]. Despite this, there are signs at both a strategic and practical level that policing in Scotland is shifting towards a more public health-oriented approach [24, 38, 47, 48]. The 2012 ‘Police and Fire Reform Act’ (Scotland) laid out a renewed purpose for policing: ‘To improve the safety and wellbeing of persons, localities and communities’ [49]. The act, through its focus on community and individual wellbeing, and on a multi-agency approach to addressing social issues, seems to provide opportunity for a shift in the focus and purpose of policing towards a more firmly public health-oriented approach [38]. Additionally, in 2020, Police Scotland launched a Drug Strategy which ‘aims to adopt a public health approach to the policing of drugs and prioritises prevention, alongside enforcement’ [38]. The recent extension of police discretionary powers in Scotland is in keeping with such strategic developments, as officers can now issue Recorded Police Warnings for Class A, B and C drugs enabling them to utilise discretion and provide a recorded warning without the individual found in possession of the illicit substance being charged with an offence [50]. Nonetheless, the extension of Recorded Police Warnings has limitations, including in relation to an individual’s history of possession offences.

This shift in the strategic aims of policing in Scotland can be seen as part of a wider policy and political shift which includes a clear recognition of drug use as a public health issue by the Scottish Government and, relatedly, the establishment of a Drugs Death Taskforce in 2019 [31]. Despite these strategic shifts, research warns of a ‘gap between policy and practice’ [38]. Indeed, despite extensive reform of stop and search in Scotland [51], little attention has been paid to its use and impact on PWUD [33]. This is despite the fact that a significant majority (76% based on the last quarter of 2021) of searches in Scotland are conducted on grounds of possession of drugs [52].

In contrast to England, where local policing divisions retain a higher degree of operational autonomy over local policing arrangements [38, 48], policing in Scotland was centralised in 2013 through the creation of a unified police service, Police Scotland. In England, divisional autonomy over policing has facilitated the development of localised diversion schemes [24], vocal support for harm reduction approaches from high-level local police officials in some areas [47, 53], and examples of local divisions working collaboratively with harm reduction services such as drug checking [1, 10]. In Scotland there are concerns that the centralisation of policing may limit divisional autonomy in relation to local policing practices (e.g., the policing of drugs). Nonetheless, the policy and legislative context in Scotland can enable change at a national level, as evidenced by the recent pilot [54] and subsequent roll-out of police carriage of naloxone to all officers across Scotland. Such institutional differences provide an important point of contrast in relation to how levels of local autonomy may shape the development of agreements between police, drug checking services and other relevant stakeholders.

Although there is a substantial body of evidence demonstrating that policing practices deter engagement with harm reduction services [6, 55, 56], there is scant attention to this within the DCS literature specifically [6]. While studies have highlighted criminalisation as a key concern for PWUD [12, 14, 57], there have been few studies focusing on policing practices outside of DCS and how such practices impact on engagement. Further, there have been no studies detailing the perspectives of police and how they interpret their role in relation to the policing of DCS and surrounding areas. This paper addresses this substantive gap and reports on the perceptions of police officers in relation to the proposed introduction of drug checking in Scotland.

This paper focuses on interviewee discussion of the challenges concerning the policing of people entering or leaving the service and of the surrounding area. Therefore, a number of relevant research questions were explored. Firstly, this paper seeks to explore police officers’ overall perceptions of the implementation and operation of DCS in Scotland. Secondly, it seeks to understand what police officers perceive to be the main challenges, and what they felt would be the best approach, in relation to the policing of DCS and surrounding areas. Lastly, this paper explores police officers’ views on the criminalisation of personal possession more generally, and how this relates to DCS.

Challenges in relation to the policing of DCS clients were chosen as the focus of the paper, as DCS in Scotland will likely operate with a Home Office Licence. The Home Office are a UK government department with responsibility for the granting of possession and/or supply licenses for controlled substances. Such licenses would provide legal exemptions for the services to handle and test controlled substances within stringent guidelines. However, such licences provide limited legal protection for clients attempting to access or leave the service. Additionally, it is important to note that the findings of this paper focus not solely on practical issues surrounding the policing of DCS, but also on how the current legislative and policy landscape, and police officers’ perceptions of such issues, intersect with the implementation and operation of drug checking. It is important to view the operation of DCS within this broader context as a means of better illuminating opportunities and challenges to move towards a more public-health oriented approach to drug use, which would have important implications for the engagement of PWUD with DCS.

## Methods

This paper reports on semi-structured interviews drawn from a larger project aiming to inform the implementation of DCS in three Scottish cities: Aberdeen, Dundee, and Glasgow. In total, 43 semi-structured interviews were conducted with stakeholders across three groups: professionals (including police, NHS and third sector (not-for-profit) staff); people who use/used drugs (in last 12 months); and family members of people who use/used drugs (in last 12 months). Ethical approval for the study was granted by University of Stirling’s General University Ethics Panel (GUEP; paper 0562).

This paper presents the views of the 10 police officer participants towards the implementation and operation of drug checking, given the importance of policing to DCS. Interview schedules were piloted with the professional stakeholder group to receive feedback and make any necessary changes. Specific questions relating to the potential legal and policing arrangements surrounding DCS were added to the schedule and asked only to police participants to ensure that discussion was relevant to the concerns and expertise of the participant group.

### Participant recruitment

Participants were eligible if they were on active duty in Police Scotland and worked in Aberdeen, Dundee, or Glasgow. Active duty in these locations was chosen as an eligibility criterion as these are the three cities where implementation of DCS is being proposed and worked towards. As noted, this paper is part of a wider research project looking to inform the implementation of these services. Therefore, it was felt that participants based on these areas would provide important knowledge about potential barriers and facilitators to implementation and operation. The inclusion of police participants as a stakeholder group in the study sampling frame was determined using a ‘selective sampling’ strategy [58] to identify stakeholder groups most relevant for ‘informing professional practice and program decision making’ [59] around the implementation of DCS. Police participants were informed of the study by an email providing relevant information. The email was originally sent to points of contact in managerial positions in each division and was subsequently circulated around frontline officers. Effort was made to recruit those with a wide range of views on drug checking by stressing in the recruitment email that the research team wanted to hear from those who both supported, and those who had reservations about, drug checking.

Once participants had specified their interest in taking part, they were provided with a participant information sheet and an opportunity to ask any questions. In addition, the researcher (DF/WM) explained the study aims prior to the interview. Written informed consent was provided prior to each interview. All interviews occurred over the telephone with DF/WM, lasted an average of 52 min (range 28–85 min) and were audio recorded. After each interview, participants were provided with a debrief sheet by email which outlined further information about the study. Post-interview memos were taken to enhance reflexivity and ensure that emerging issues and concepts were adequately captured to inform data analysis.

### Data analysis

Data were transcribed by a professional transcriber in full and analysed in NVivo12 (QSR International Pty Ltd., 2020). Transcripts were coded inductively using Thematic Analysis to identify emerging themes [60]. The initial coding was conducted by one researcher (DF). The codes were then discussed in depth with another member of the research team (HC), with anything that was unclear or could have different interpretations highlighted and adapted. After coding several transcripts from all stakeholder groups, the initial coding framework was sense-checked and discussed by other members of the research team (HC, TP, WM). Once the framework had been discussed and agreed upon by these authors, the remaining transcripts were coded using the framework by DF and WM. Additions and refinements were made throughout the coding of the remaining transcripts, with both researchers meeting regularly to discuss the level of fit between the coding framework and data. After all transcripts had been coded, the research team returned to the codes that were specifically relevant to the policing and legal challenges surrounding drug checking. As such issues emerged as a major consideration in discussions with wider project stakeholders, a decision was made to focus on the perceptions of police participants for this paper.

## Findings

Of the 10 police interviewees two identified as women and eight as men, which is broadly in line with the demographic profile of Police Scotland staff [61]. Participants were based on Aberdeen (*n* = 5), Dundee (*n* = 2), and Glasgow (*n* = 3), had a range of ranks including Constable, Sergeant, and Inspector, and described varying years of service ranging from a few to over 30 years.

The findings are presented in three themes: general perceptions of DCS; shifting culture towards public health-oriented policing; and issues and challenges surrounding the policing of DCS. Within these, a number of sub-themes were identified (Table 1).

**Table 1:** Themes and sub-themes

Theme 1: General perceptions of DCS
Theme 2: Shifting culture towards public health-oriented policing
2a: The perceived failure of enforcement-based practices
2b: Increasing awareness of social and structural drivers of drug harm
2c: Limits to the shift in public health policing
Theme 3: Issues and challenges surrounding the policing of DCS
3a: Preference for legislative change or national strategic guidance
3b: Policing the area surrounding a DCS
3c: The role of officer discretion

### General perceptions of DCS

The majority of participants (*n* = 8) expressed general support for the implementation and operation of DCS in Scotland, while highlighting concerns around the policing of such services. Several participants noted that people were always going to use drugs, regardless of enforcement, and that, therefore, there was a need to provide people with information to minimise risk: ‘*I think we need to accept the reality that people will take drugs and its just educating them as to what they are putting in their system*’ (Police#1)*.* The concepts of safety and preservation of life recurred in discussions, with participants using these to square their role enforcing drug laws with support for drug checking. As expressed by one participant, interventions such as drug checking were increasingly seen as defensible by senior management within the police, as use of such services would indicate that an individual wants ‘*to keep themselves safe*’ (Police#3).

Participants discussed the volatility of the drug market, making the provision of information to PWUD increasingly important for protecting life. The variation in strength and content of ‘street benzos’ (novel benzodiazepines) was seen as particularly concerning. Owing to these factors, and the resultant high level of drug related deaths in Scotland, participants often described a sense of moral imperative to explore alternative means of addressing the situation. As expressed by one participant, not implementing harm reduction interventions to address the level of drug related deaths, in their view, amounted to ‘*allowing people to die where we could have intervened*’ (Police#6).

Although most participants were supportive of drug checking, some expressed significant reservations. One participant discussed having very limited personal knowledge of drug checking in relation to how and where it would operate. However, they described the concept as intuitively ‘*odd*’ (Police#8). Whilst noting that in an ideal world they would ‘*like all drugs taken off the street*’, they acknowledged that a drug free society was not possible and displayed understanding of the underpinning logic of DCS: ‘*to reduce the harm of someone taking something which is going to cause serious harm or death*’ (Police#8). However, they described having significant reservations around the operation of DCS, and the role of the police within it:

*Are we saying the police are openly welcoming people to come into a building when drugs are in their possession to have it checked? At which point they are committing a crime immediately*. (Police#8)

In relation to support amongst the organisation more generally, participants described a perceived willingness amongst high-ranking police officials to work collaboratively with harm reduction services such as drug checking. Participants also generally believed that support for DCS would be fairly widespread amongst colleagues in their respective local divisions. For example, one participant highlighted that their local division would welcome the implementation of drug checking as an ‘*extra tool to try and keep people alive and safer*’ (Police#5). Another expressed the view that there was likely to be varying reactions to drug checking across departments, due to differing remits and cultures. They elaborated that their department would potentially be very receptive to the implementation of drug checking:

We’ve got the harm reduction assertive outreach part, that would sit nicely. We have just announced that we are having a harm reduction sergeant, that will be their specific role. So, by all means, I think it would sit very well here and would be welcomed. (Police#6)

However, not all shared the view that drug checking would be similarly supported. While noting a willingness from higher level police actors to support such interventions, one participant stated that drug checking would struggle to receive buy-in from local police teams, describing this as a ‘*hard sell*’ (Police#8).

Despite most participants noting that there would likely be reasonable support for DCS within the police at both national and local levels, many also felt that the police should not offer a strong opinion in support for such controversial services. Indeed, participants tended to stress their perception of the police as politically neutral, drawing their legitimacy from public confidence and through the process of ‘*policing by consent*’ (Police#8). Expressing vocal support for such an intervention could, in their view, harm public confidence in policing:

I think it’s a dangerous thing for the police to offer an opinion because you know the police are…the grey man sitting in the corner. If the police offer and opinion and say ‘use this service’, then there could be a public perception that the police are encouraging people to take drugs. So, the police would really need to stay out of the politics, really need to stay in the background and leave it up to a public discussion. (Police#1)

Participants tended to suggest that police could tacitly support DCS through not interrupting their operation, thus enabling engagement. However, as will be explored in more detail below, they also noted the need for high level actors to provide them with ‘cover’ in doing so, so that local officers were able to justify their practices to the public by pointing to guidance from relevant stakeholders. Examples of such potential cover included legislative change, directives from the highest-ranking police officials, or national guidance from the Crown Office and Procurator Fiscal Service, Scotland’s prosecution service.

### Shifting culture towards public health-oriented policing

Many participants described support for DCS, both at an individual and a perceived organisational level, as part of a broader shift in policing culture away from support for enforcement-focused practices towards an openness to adopt a more public health-based approach. For example, one participant provided an account of this shift during their approximately 30 years of experience in policing:

When I joined, it was purely enforcement. There was very little, if any, thought given to a public health approach to this problem, and very little given to the welfare of those involved. It was purely enforcement and that has changed almost 180 degrees in the time that I’ve been in the police. (Police#4)

Participants described both indicators of, and factors perceived to be shaping, this shift in culture, which are described below as sub-themes. Whilst such discussion related to the intersections and potential tensions between public health and policing of drug laws more broadly, rather than to challenges around DCS specifically, it is worth outlining participants’ perceptions of such issues. Such discussion was a major feature of the data and served as a crucial contextual backdrop for how participants were able to square their support for the implementation of DCS with their role enforcing drug laws. Such issues speak to the complex process by which the ‘law on the books’ (i.e., criminalisation of drug possession) is mediated through a range of complex organisational, interpersonal and cultural factors [27, 38, 47].

#### The perceived failure of enforcement-based practices

Several participants described enforcement-led approaches as ineffective and unsustainable. As noted by one participant ‘*an enforcement-led approach doesn’t work for folk that are simply using drugs*’ (Police#3). Many participants described a belief that a public health approach to drug possession had proven to be successful in other countries and should be adopted in a Scottish context. Some described how viewing drug use through a ‘*health lens*’ had the potential to increase the security and wellbeing of communities by reducing levels of drug related death and harm, and related social costs:

Using a health lens, it’s better for everybody. It’s better for communities, you know? You don’t want to be a neighbour who has, you know, got somebody next door that dies. That’s not a good feeling and it brings an area down. If we can prevent that, however we prevent it, then it makes these communities better places to live, better places to work, better places to visit. (Police#6)

Indicative of a growing openness to move away from enforcement-based approaches, several participants voiced support for changes to legislation around drug possession and use, ranging from diversion and depenalisation to de jure (legislative) decriminalisation. In relation to diversionary schemes, one participant stated that people who use what they termed ‘*hard*’ drugs could be offered treatment as opposed to being prosecuted (Police#2). Others expressed the view that simple possession up to a threshold amount should be lifted out of the criminal sphere entirely and instead be addressed by health and social services, without coercion or threat of prosecution:

Like if you have someone with one wrap of heroin on them, you know, is that something that the Government and the [police] could look at and say ‘OK we will take possession of that and we will mark it for destruction with no further proceedings’? It frees up court time, it frees up all this paperwork and stuff like that and then you could signpost these people to drug treatment centres. (Police#9)

#### Increasing awareness of social and structural drivers of drug harm

Participants described the perception that daily policing practice was increasingly related to dealing with welfare and mental health issues, rather than addressing offending behaviour. They frequently described the police as ill-equipped to address such issues, creating a perceived imperative to explore means of ensuring that such issues were instead managed by appropriate social and health services. It was felt that this would enable the redirection of police resources towards criminal offences which threatened community safety and security. Participants further described how exposure to the social conditions of drug use and the lives of PWUD had led them to better understand that important drivers of ‘drug problems’ were often environmental and structural, and beyond the control of the individual, thus highlighting the limited impact of law enforcement on such issues. One participant highlighted how their personal exposure to the social suffering of affected families and communities had caused them to shift towards viewing drug use as a public health issue: ‘*I’ve probably been to, in the number of hundreds of drug deaths, so I’ve seen the impact on families, I’ve seen the impact on these people*’*.* (Police#9).

Although participants described an increasing understanding of social issues and their links to drug harms, few participants discussed the role of policing practices in exacerbating such dynamics and the harm experienced by marginalised individuals. Only one participant discussed the harm and stigma incurred by policing practices such as stop and search within a ‘*fairly embedded culture*’ which was slow to shift:

If you have got somebody who is a drug user, we will stop them on the first grounds, we will search them and deal with them on the street, you know? And that creates stigma because the half a dozen people that walk past the police officer stopping that person in the street are going to say ‘Oh that’s such and such, what’s he been up to? Just another drug user’. (Police#3)

#### Limits to the shift in public health policing

Despite participants describing a shift in policing culture, there are important caveats to consider. Some participants noted that legislation criminalising personal possession limited the capacity of officers to shift towards a public health approach in practice. This was illustrated in the tension, often inherent in participants’ descriptions of their roles, between two, seemingly contradictory, functions in dealing with drugs—criminalisation of drug possession, and support for PWUD:

Overall, we have two main roles, the police, with regards to substance use. One is the welfare of any people who might be using illegal drugs of any kind. That’s our overarching job description I would suggest for any person in the community…and the other one is obviously enforcement around the Misuse of Drugs Act and in dealing with anybody that breaks that piece of legislation. (Police#4)

Whilst often tentatively supportive of legislative change to decriminalise personal possession, or at least to reduce criminalisation of PWUD, the tension between practices of support and practices of criminalisation was often an uneasy one. Some participants referred to the idea that enforcement was an important aspect of a public health approach, rather than a contradictory and damaging practice, justified on the grounds that: ‘*drugs are dangerous and against the law for a reason*’ (Police#1)*.*

Participants also acknowledged that the culture within the police was fragmented and was changing unevenly, reporting that many officers were still supportive of enforcement-led approaches. This was demonstrated by one participant’s discussion of the police carriage of naloxone pilot in their local division which ‘*generated quite a lot of debate internally*’ (Police#4). Participants highlighted the need for education and training to help shift cultures but highlighted that this process would be generational.

### Issues and challenges surrounding the policing of DCS

Participants outlined challenges around the policing of DCS within the current legislative framework. They noted a perceived need for either legislative change or national strategic guidance to support local divisions and officers in shifting their practice in the required ways. Participants also discussed different potential approaches to the policing of DCS and the surrounding areas.

#### Preference for legislative change or national strategic guidance

Participants expressed a strong preference for either legislative changes or national strategic guidance explicitly outlining how the area surrounding the service should be policed. The ideal option for participants seemed to be legislative change to enable policing practices to be more aligned with public health goals, although it was acknowledged that the power to alter drug legislation lay with the UK Government. Noting the potential challenges of securing legislative change, some participants discussed the potential for what might be termed ‘national strategic guidance’ to inform the policing of DCS. This was seen as potentially taking different forms. Most commonly, guidance from the Lord Advocate/Crown Office and Procurator Fiscal Service (prosecution service) was seen as a potential means of providing police with clear guidance and support to employ more public health-oriented practices:

The Lord Advocate would be in the position to say that anyone who is going to or from a drug checking service is not responsible criminally. They would have the power to be able to do that and we would act on those guidelines. (Police#8)

Other potential national guidance discussed included statements issued from senior police officials and Scottish Government. Having clear national guidance, as opposed to relying solely on informal agreements between local divisions and DCS, was seen as important in providing sufficient support for local divisions and officers to alter their practice. One participant described drugs as a ‘*massive political hot potato*’ and noted that being able to point to clear guidelines from those with the relevant authority around policing practices and processes would help police defuse the political element and justify practices to local communities (Police#5). This was echoed by another participant, who described the need for police to be protected from ‘sensationalising’ news coverage and negative public attitudes:

Well it could be argued that if you are passing the drug checking [service] and you see a known user walking towards it… you know they are going there for a particular reason. So, if you do nothing is that a dereliction of duty? Well yeah it could be, and all you need is a member of the public to film it and say you know… ‘I told him what that was and the officer did nothing. They knew that that person had drugs on them’… and then you are back to your red top papers sensationalising it where actually we could be saving a life. (Police#6)

Others felt that having clear national guidance would reassure individuals planning to use the service that they would not be placed at risk of harassment or arrest for trying to access the DCS. However, even with national strategic guidance in place, there are significant limitations in the extent to which individuals will be protected in the context of criminalisation. As expressed by participants, for a number of reasons it is very challenging to provide a ‘*complete blanket*’ reassurance to clients that they will be protected from criminalisation (Police#7). Despite such challenges, it was generally felt that relying solely on local informal agreements would leave clients too open to discretionary, inconsistent policing practices.

#### Policing the areas surrounding a DCS

Participants described concerns around the concept of a ‘tolerance zone’ or ‘boundary agreement’ around DCS. A tolerance zone is, broadly, an agreement on how the area surrounding a DCS would be policed [29, 55, 62]. The details of how such spaces would operate is not well developed and would require careful consideration. Although ‘tolerance zone’ or ‘boundary agreement’ are the terms commonly used in the literature, this paper will herein refer to such arrangements as an ‘enhanced support zone’. This term has been developed in a Scottish context due to its being seen as a more acceptable term amongst a wide range of stakeholders involved in dialogue on the policing and legal challenges facing proposed DCS. Participants tended to interpret the concept of enhanced support zones in two distinct ways: as an arrangement based on limiting police presence in the vicinity of the service and/or limiting the scope of police stop and search in cases of suspected personal possession; or as an agreement to enable police not to charge someone for personal possession, below a threshold quantity [28, 63], within a specified zone.

Although acknowledging that a heavy police presence and use of stop and search in the vicinity of DCS may act as a deterrent to engagement for prospective clients, participants generally expressed discomfort with any arrangement seeking to limit police presence or stop and search. Participants noted a perceived potential for people to take advantage of such arrangements, leading to increased crime and social disorder in the surrounding area:

The challenges are that you might draw in the wrong type of people within that area and you might encourage you know drugs misuse within that area… or drug dealing. People might think they can take advantage of that. (Police#1)

The larger the size of the zone, the more it was perceived as a risk to social order and community security. However, not all participants felt that such arrangements would increase social disorder and crime in the local area. One participant drew on the example of community pharmacies which provide clients with opioid substitution therapy (OST) and injecting equipment provision (IEP) and have established arrangements in relation to policing practice, highlighting that there are ‘*very few incidents*’ outside pharmacies (Police#4). Although the example of a pharmacy is different from a defined and formalised support zone, it does highlight that policing arrangements concerning DCS can learn from established practices in relation to the policing of other harm reduction interventions.

A related concern discussed by participants was that reduced police presence, or level of stop and search, would curtail the police’s capacity to respond to public concerns, whether real or perceived, around social disorder and crime in the vicinity of the service. Participants felt that this could damage police and public relations and thereby erode the perceived legitimacy of the police. Any restriction on the capacity of police to respond to community concerns was seen as potentially problematic. One participant highlighted, for example, that the police are ‘*intelligence-led*’, making it difficult for them not to respond to intelligence from the public around perceived criminal behaviour taking place:

It could be a completely false perception but if [the public] say ‘there is drug dealing going on there, that person is drug dealing’, we are sort of put in a situation where even if there is an [enhanced support zone], we are intelligence led, so if there is intelligence that there is drug dealing going on […], we are in the position that we have to go and look at that and speak to somebody. (Police#7)

It should be noted that discussion around an enhanced support zone was, at times, based on a degree of misunderstanding around how such an arrangement would likely work. Two participants conflated simple possession with people consuming drugs in public spaces and noted that the police would be unable to intervene. They did not discuss the range of responses which could be available to them in such a situation, including non-criminalising, welfare, and dialogue-based responses [29]:

If you had a [support] zone, they would tell the police that they no longer have the power of search under the Misuse of Drugs Act. So, you are then left in the crazy situation where police officers, or anybody else, are left looking at somebody shooting up drugs, swallowing Valium, doing whatever it is they are doing, and then we can only interact when it looks like they are in danger of harming themselves? (Police#4)

Close working relationships between DCS and police were described as important to managing the relations between service, people who use the service, wider community, and police:

It’s a sort of three-way thing where the police would need to liaise with [the service] and also, you know, the general public, and if any issues are being raised by the general public the police would need to let [the service] know, and vice versa. If you think you will get complaints from the public about your service, then you would need to feed that back to the police. (Police#1)

As noted, the other way in which participants often envisioned an enhanced support zone operating was for personal possession to be ‘decriminalised’ or ‘depenalised’ (either formally through legislative change or through expansions to police discretionary powers), up to a threshold quantity, within a specified zone around the service. This was seen as a more feasible approach than relying on a reduction in police presence and stop and search practices, although participants still discussed challenges in relation to such an approach. It was highlighted that police would need to operate with clearly defined possession limits, beyond which possession would still be considered a criminal offence. A couple of pills or a small amount of powder were described as potential threshold limits, with an emphasis on a small amount necessary for the testing process. One participant described the need to ensure that people were aware of these limits so that ‘*everybody is clear about what they can get away with and what they can’t get away with*’ (Police#1).

While most participants discussed an enhanced support zone as a space with formalised changes to police practice in the ways outlined above, some described the potential for a less formalised approach. Such an approach was described as being based on an understanding around the policing of DCS which should be aligned with the desire for people to engage with the service, drawing on established practices regarding community pharmacies and IEP sites. Although police are aware that people receiving IEP, for example, would likely be in possession of drugs, they do not generally target people accessing this service as it is agreed that such practice is not in the interest of public health. One participant explained that agreement around DCS would need to be based on similar principles:

It just needs to be explained to the people using it that the police aren’t looking at this as somewhere that they are going to be watching with binoculars, standing outside, knowing everyone’s details. But that they are aware that there is harm reduction going on here and that it actually fits their expectation of what they can do to reduce harm in the community. (Police#7)

The integration of drug checking in existing harm reduction services was therefore seen as positive by enabling police to refrain from targeting people entering or leaving the service, as people could be accessing the service for various reasons—limiting probable cause for stop and search. Although such an approach would have less defined rules in relation to policing of a DCS, participants highlighted that they still felt it would require national strategic guidance and support from high level actors, as opposed to solely localised agreements.

#### The role of officer discretion

Participants highlighted that, short of legislative change to decriminalise personal possession, people accessing the service may still be vulnerable to harassment, surveillance, and being charged when attempting to access the service. Participant discussion of police officer discretion and local policing cultures, and the consequent impact of such factors on the policing of PWUD, particularly those who could be considered marginalised, highlights the potential risks faced by individuals trying to access DCS, even in the event of a national agreement around the policing of these services. One such issue raised related to police using DCS as an ‘*avenue*’ to identify people ‘*wanted on warrants*’ for other offences (Police#9). Relatedly, the potential for police to use DCS to identify and target suppliers was discussed, with one participant acknowledging that ‘*it won’t be the big boys who are doing this, it will be the runners, you know, probably the users or young people on their way down that route*’ (Police#6). Some participants highlighted that, given officers would always be able to find ways around agreements in relation to policing of DCS, there would be a need for local divisions and officers to buy in to the concept of drug checking, and to understand why such practices are counter-productive from a public health standpoint. It was highlighted that there may be a need for a ‘*real shift in culture to make it work efficiently*’ (Police#9).

Interestingly, despite the examples outlined above of how police could circumvent the spirit of agreements around the policing of DCS, the role of discretion in daily policing was often not an explicit feature in participant accounts. Participants instead described Police Scotland as a ‘*structured*’, ‘*disciplined*’ and ‘*hierarchical*’ organisation: ‘*You know it’s a disciplined service and we will basically do what we are told*’ (Police#10). Due to this perception frontline policing was often seen a process of straightforwardly enforcing the ‘law on the books’ [27]:

There is some legislation that we’ve got more leeway than others. But we cannot ignore people in possession of drugs. We cannot, we can’t do it whether we want to or not. (Police#4)

The absence of explicit consideration of the role of discretion was apparent in relation to discussions of stop and search practices, and when it is deemed necessary to subject someone to this process. Some participants implicitly described utilising various discretion-based judgements to determine whether there was probable cause to stop and search someone for suspected possession:

So, you are speaking to somebody, and it looks as if they are already under the influence and as if they’ve already consumed some drugs, that would be the way you’d look at it and you’d be like, you know, ‘Are you okay? Why are you acting the way you are acting just now, is it because, you know, is it a mental health issue, is it a drug substance issue?’ You know you would see the froth around the mouth, certain drugs that were taken would have like a distinctive… not froth it’s more of a white, you can see a white ring around the mouth, you’d use that, ‘You look like you’ve taken drugs and we are going to search you to check you don’t have more on you at the moment’. But aye it’s, that’s kind of the way I look at it, the justification for stopping and searching somebody. (Police#2)

Another participant highlighted the role discretion plays when deciding to stop and search someone. They described knowing that someone was accessing a DCS as probable cause for stop and search, highlighting how, in the absence of robust agreements around the policing of DCS, police officers who are less supportive of public health approaches can apply their discretion in ways which are detrimental to harm reduction objectives. Another participant felt that ‘*discretion*’ was not an appropriate term to use when describing the decision to stop and search someone as ‘*you must have probable cause to search somebody*’ (Police#9)*.* However, they noted that this may be based on judgements such as the appearance of a person as ‘*technically drug users present a certain appearance*’. Participants also described how directives from higher ranking officials can shape the practice of stop and search, and how discretion is exercised in relation to such factors. They described a past instance where a Chief Constable had applied pressure on the organisation to increase instances of stop and search:

We had a time when we had a Chief Constable who just basically just…was encouraging stop and search constantly, you know, everybody has to stop and search because they wanted targets etc… they wanted to reduce crime. (Police#1)

These comments highlight that, while decisions to stop and search were described as being based on the concept of ‘probable cause’, such decisions were implicitly described by participants as shaped by both individual officer discretion and institutional factors at local and national level. The description of appearance as a deciding factor in whether to stop and search someone is particularly demonstrative of the role of discretion, and potential for discrimination, in stop and search practices.

## Discussion

Most participants indicated a general support for the introduction of DCS in Scotland given that they enable people to potentially reduce risk and harm in the face of a volatile and unregulated drugs market. This stance was underpinned by a perception that people would continue to take drugs, and that enforcement-based practices in relation to personal possession were ineffective and exacerbated harm for PWUD. Participants were able to resolve tensions between their role as enforcers of drug laws with support for DCS by highlighting drug checking as a tool for increasing safety at a community and individual level. Such findings highlight that frontline police officers do not merely enforce legislation but, rather, actively participate in a process of interpreting and implementing the law, mediated by a range of complex, and sometimes competing, demands, interests, values and beliefs [27, 47, 64]. Participants often strongly identified with their role in preserving life and working to ensure safer communities, with DCS seen as having the potential to contribute to these goals. Such interpretations create avenues for policing to be more aligned with public health goals, highlighting the potential role of supportive frontline police officers and local divisions in pushing for institutional change through challenging established ‘cultural scripts’ on the role and function of policing in relation to drugs [47].

There have been examples of local policing divisions taking such approaches and using discretion and autonomy to provide support for harm reduction services by interpreting their role in line with concepts of protecting and enhancing safety and wellbeing [47, 48]. The capacity for local divisions to do so in a Scottish context is complicated by the centralised nature of the police service which seems to limit the perceived autonomy of local divisions. However, the unsanctioned mobile overdose prevention centre which operated in a van in Glasgow for 9 months (September 2020–May 2021) may indicate some willingness and capacity amongst local police to utilise discretion in the interests of harm reduction [65]. Although the service did present challenges for police, it operated without being shut down.

Participants described a general perception of a cultural shift within Police Scotland, at both local and national level, away from a focus on enforcement-based practices towards an openness to viewing drug use as a public health issue. This echoes the wider literature which suggests that such shifts are increasingly common internationally, albeit slowly, unevenly, and often with significant limitations [24, 28, 30, 48, 66]. This is also in line with recent steps in Scotland towards a more public-health approach to policing, including the aforementioned police carriage of naloxone pilot [54] and subsequent roll-out to all officers across Scotland, and the extension of Recorded Police Warnings to Class A drugs.

Importantly, participants described being supportive of legislative change to either increase the provision of diversionary activities, depenalise drug offences, or to fully decriminalise personal possession up to a threshold quantity. However, it is important to note the distinctions between these different approaches [28]. Whilst some endorsed removal of possession penalties from the Misuse of Drugs Act 1971, others favoured retaining enforcement as a lever in diversionary schemes [67]. Regardless of the limitations concerning participant willingness to embrace decriminalisation of personal possession, there was a general perception that law enforcement was limited in addressing the ‘drugs issue’. This was indicated by participants describing a growing awareness within the organisation that the drivers of drug related harm were often socio-structural, and therefore best addressed by partner agencies in the health and social fields. Such shifting logics point to the potential for building shared ground around addressing drug use and harms primarily through a public health lens. However, owing to the small sample size of the present study, further research is required to explore the extent of support for public health-oriented policing amongst frontline officers in Scotland.

Despite support for the implementation of DCS, all participants expressed concerns regarding policing arrangements of the service and surrounding areas, primarily in relation to the establishment of enhanced support zones around services. Maintaining community order and responding to concerns of local residents was described as a key function of policing, and one that could potentially be in tension with the desire to enable access to DCS. Research on police presence around supervised injection sites in Canada has demonstrated the tensions and challenges around these functions, for example in responding to concerns around community order yet also enabling access to supervised injecting sites in the interest of harm reduction [18, 55, 62]. Policing responses to services, even amongst those which exist within the same legislative framework, differ according to the ‘unique implementation contexts’ of services, including whether the surrounding area is undergoing gentrification and the extent of open-air drug scenes [18, 55]. This highlights that police support-in-principle for harm reduction services may not always translate into support-in-practice, depending on the level of pressure on police to respond to community concerns [6, 8, 55]. Participants in our study discussed the use of police liaison officers to mediate potential tensions between communities and DCS. This echoes research on supervised injection sites which found that close dialogue between police and services, and dedicated police liaisons, can help mediate challenges and tensions [62].

As well as limitations to police presence and/or stop and search in the vicinity of services, participants also discussed the potential for effective decriminalisation, or at least depenalisation (either through extensions to police discretion or changes to legislation), of personal possession within a set boundary around DCS. Such arrangements were conceptualised as having strict ‘threshold’ quantities above which someone would still be subject to being charged, with an emphasis on people only carrying the minimum amount required for testing. This raises questions around how much protection would actually be afforded to people who access DCS under such arrangements. Particularly where drugs such as benzodiazepines are cheap, and often purchased in large quantities, it is likely that people who access DCS may be carrying larger quantities than the minimum threshold amount.

Although participants were skeptical of the feasibility of a formalised enhanced support zone around DCS, international examples highlight that such arrangements are possible with no evidence of increasing social disorder in the vicinity of such services. Drug consumption rooms in Copenhagen present an example of such agreements [29]. In this example, directives indicate that ‘police should not “normally” charge people for possession of illicit drugs for personal use in the “immediate vicinity” of drug consumption facilities’, with the definition of ‘immediate vicinity’ collectively decided by local police and the municipality [29]. Research suggests that this agreement has facilitated a shift in policing practice by increasing the options available to officers in dealing with public disorder or possession concerns [29]. A further example lies in the legal arrangements surrounding DCS in the Netherlands. Whilst not based on a geographically-defined enhanced support zone, as previously noted, such services operate under agreement with the public prosecutor that people will not be prosecuted for possession when trying to access the service [15]. This is supported by a public health approach to drug use in the Netherlands more generally. Drug policy is the preserve of the Ministry of Health, with prosecutions for possession of drugs relatively rare [15, 42].

The role of police officer discretion was an important finding in relation to the policing of DCS. Although police in Scotland undoubtedly exercise discretion in terms of where, when, how and who to police, the extent of perceived discretion varies and there are differences between England and Scotland given that police in Scotland must refer cases to the Crown Office and Procurator Fiscal Service (prosecution service) for prosecutorial decision making. The extension of the Recorded Police Warning system in Scotland will undoubtedly affect the extent of officer discretion in relation to personal possession but there is currently a lack of data and analysis available on how this is working in practice. Participants in the present study minimised the role of police discretion in relation to enforcing drug laws, for example stating that they could not ignore people in possession of drugs. Despite this, discussion of the policing of DCS highlighted several potential uses of discretion in ways which could undermine the goals of increasing people’s safety and enabling access to the service. Examples included using DCS as an ‘avenue’ to find people wanted for other offences, placing the service under surveillance to target drug suppliers, and considering use of the service as probable cause for stop and search. Two participants also described using a person’s visual appearance as a measure of probable cause. Literature highlights that marginalised groups are particularly susceptible to being targeted on the basis of belonging to a minority and/or marginalised group, which can often be discerned by particular indicators such as physical appearance [18, 20, 68–70]. These examples of the use of discretion echo wider literature on the ways that discretion can be used to undermine public health objectives as well as support them [28, 29, 66]. Such issues thus present potential challenges under any set of arrangements short of de-jure decriminalisation of personal possession.

### Implications for policy, practice and research

There is a need for pragmatic arrangements to be swiftly put in place in relation to the policing of DCS to enable implementation during a public health crisis in Scotland. The purpose of policing in Scotland, enshrined in legislation, is to enhance the wellbeing and safety of individuals and communities. Further, the drugs portfolio in the Scottish Government currently sits with the health directorate rather than criminal justice, and the Government’s most recent alcohol and drug strategy emphasises a human rights-based approach. For these reasons it is hoped that multi-stakeholder agreements may be developed in line with national priorities and policies, to provide clarity and support to local police and reassurance to people when using the proposed DCS.

Informal agreements between local divisions and DCS may well form the basis of these arrangements. We would suggest that national strategic guidance from relevant stakeholders could provide support for local divisions to align their practices in supportive ways. There are, however, significant limitations in the extent to which such arrangements provide guarantees of protection for people accessing the service. While recognising the complexity and challenges in securing legislative change, there is a need to move towards decriminalisation of personal possession in order to meaningfully shift towards a public health approach to drug use and related harms. While Scotland is not able to put in place de-jure decriminalisation given drugs policy is reserved to the UK government, there is a need for careful consideration of the available means of moving towards a less criminalising approach to drug possession, including substantial extension of police discretionary powers (such as further extensions of Recorded Police Warnings). Finally, close liaison between local police officers and DCS can help build and maintain supportive relationships and proactively address emerging challenges and tensions. The establishment of a lived experience/police interface may help further facilitate dialogue on key issues and challenges.

As harm reduction services such as DCS are established in Scotland, there will be a need for ongoing research to explore how agreed approaches to the policing of such services are enacted on the ground and experienced by PWUD. It will be crucial for such research to assess the level of concordance, or otherwise, between agreed policing strategies and frontline policing practices. There is also a need for research on how the extension of Recorded Police Warnings in Scotland impacts policing practices in relation to possession of drugs.

### Strengths and limitations of the study

This study captured the views of policing participants across three Scottish cities in a range of roles and varying levels of seniority. It is the first study, to our knowledge, to explore the views of police officers regarding DCS. Exploring this topic provides insight into the potential tensions and challenges facing the implementation and operation of DCS, as well as presenting potential points of leverage to build a shared understanding of the benefits of harm reduction and of public health-oriented policing. Whilst these findings relate specifically to Scotland, there is potentially transferable learning for other jurisdictions. There are two primary limitations. Firstly, findings are based on interviews with a small, self-selecting sample of police participants which means that it is not possible to know the extent to which participant perceptions of drug checking and public health-oriented policing are representative of the wider culture within Police Scotland. It may be the case that the sample is biased by self-selection, as those who are more supportive of harm reduction approaches might be most willing to put themselves forward for interview. However, although participants expressed general support for DCS, they also expressed concerns around the policing challenges in relation to such services. The findings are therefore able to provide important insight into the key considerations of legal and policing challenges in relation to DCS in Scotland. However, further research is required given the small sample size on which the findings of the present paper are based. Secondly, interviews were conducted before the extension of Recorded Police Warnings to Class A drugs. Had the interviews been conducted after this change, discussions may have highlighted how such arrangements might interact with frontline policing in relation to DCS.

## Conclusion

This paper has explored police officer perceptions of the policing and legal challenges which could be encountered if community-based DCS are introduced in Scotland. While findings indicate a general support for DCS as part of a wider organisational shift towards more public health-oriented policing, participants noted concerns and challenges around the policing of DCS within the current legal framework. Findings indicate a perceived need for careful consideration and discussion of the steps necessary to move towards a more public health approach to the policing of drug possession, one which enables access to vital harm reduction services.

## Data Availability

The datasets generated and/or analysed during the current study are not publicly available because individual privacy could be compromised if the dataset is shared due to the small sample involved and the fact that the cities and organisation are named.
